# Phylogeography and ecological niche modeling implicate multiple microrefugia of *Swertia tetraptera* during quaternary glaciations

**DOI:** 10.1186/s12870-023-04471-w

**Published:** 2023-09-26

**Authors:** Lucun Yang, Guoying Zhou

**Affiliations:** 1 Northwest Institute of Plateau Biology, Chinese Academy of Sciences, Xining, 810008 China; 2 Qinghai Key Laboratory of Qinghai-Tibet Plateau Biological Resources, Northwest Institute of Plateau Biology, Chinese Academy of Sciences, Xining, 810008 China

**Keywords:** cpDNA *trn*S-*trn*G, *Swertia tetraptera*, Haplotypes, Qinghai-Tibetan Plateau, Refugia, Phylogeographic structure, Quaternary glaciations

## Abstract

**Background:**

Climate fluctuations during the Pleistocene and mountain uplift are vital driving forces affecting geographic distribution. Here, we ask how an annual plant responded to the Pleistocene glacial cycles.

**Methods:**

In this study, we analyzed the population demographic history of the annual herb *Swertia tetraptera* Maxim (Gentianaceae) endemic to Qinghai-Tibetan Plateau (QTP). A total of 301 individuals from 35 populations of *S. tetraptera* were analyzed based on two maternally inherited chloroplast fragments (*trn*L-*trn*F and *trn*S-*trn*G). Phylogeographic analysis was combined with species distribution modeling to detect the genetic variations in *S. tetraptera*.

**Results:**

The genetic diversity of *S. tetraptera* was high, likely due to its wide natural range, high proportion of endemic haplotypes and evolutionary history. Fifty-four haplotypes were identified in *S. tetraptera*. Only a few haplotypes were widespread (Hap_4, Hap_1, Hap_3), which were dispersed throughout the present geographical range of *S. tetraptera*, while many haplotypes were confined to single populations. The cpDNA dataset showed that phylogeographic structuring was lacking across the distribution range of *S. tetraptera*. Analyses of molecular variance showed that most genetic variation was found within populations (70.51%). In addition, the relationships of the haplotypes were almost completely unresolved by phylogenetic reconstruction. Both mismatch distribution analysis and neutrality tests showed a recent expansion across the distribution range of *S. tetraptera*. The MaxEnt analysis showed that *S. tetraptera* had a narrow distribution range during the Last Glacial Maximum (LGM) and a wide distribution range during the current time, with predictions into the future showing the distribution range of *S. tetraptera* expanding.

**Conclusion:**

Our study implies that the current geographic and genetic distribution of *S. tetraptera* is likely to have been shaped by Quaternary periods. Multiple microrefugia of *S. tetraptera* existed during Quaternary glaciations. Rapid intraspecific diversification and hybridization and/or introgression may have played a vital role in shaping the current distribution patterns of *S. tetraptera.* The distribution range of *S. tetraptera* appeared to have experienced contraction during the LGM; in the future, when the global climate becomes warmer with rising carbon dioxide levels, the distribution of *S. tetraptera* will expand.

**Supplementary Information:**

The online version contains supplementary material available at 10.1186/s12870-023-04471-w.

## Introduction

The Qinghai-Tibetan Plateau (QTP), which covers approximately 2.5 × 10^6^ km^2^ or one-quarter of China, is the world’s largest and highest plateau, with an average altitude of 4500 m above sea level [1]. In the lower Cretaceous, the uplift of the QTP was caused by the collision of the Indian Plate with the Eurasian Plate [2]. Large changes have taken place in the biota of the QTP and its neighboring mountains due to the alteration in topography and local climate. Numerous species have become extinct [3, 4]. However, the southern edge of the QTP and the Hengduan Mountains region has been labeled a biodiversity hotspot [5]. In addition, a new and young biota has developed on the plateau [6, 7]. Among alpine seed plant species, 34% are endemic to the QTP [8], whereas only 2.9% of the genera are endemic [6]. Where this young alpine flora originated and how the plateau uplift and climatic fluctuations during the Quaternary glacial period have influenced their differentiation, evolution, and dispersal are the subject of continuing debate.

Using the phylogeographic method [9], the phylogeographic history of some species on the QTP and its adjacent areas has been explored recently [10–22]. However, most of these species were trees or perennial herbs, and only a few studies have concentrated on annual taxa [23, 24], and it has been established that the genetic diversity of plant species is affected by life-history traits [25]. Compared with long-lived trees, annuals, with their specific life-history characteristics, may show different levels and structuring of genetic variation. The high selfing rate [26], low mutation rate [27] and short generation time are the main characteristics of annuals that could influence the level and structure of diversity. Furthermore, the failure to reproduce due to adverse conditions in a particular year may have a strong impact on the demography of a population of annuals, and as a result, annual species may be expected to experience more bottlenecks. Research on such species is therefore necessary to increase our understanding of how Quaternary climate changes have affected the range distributions and intraspecific divergence of alpine plants on the QTP.

The history of a species in space as well as time can be revealed by phylogeography [28], however, until recently, geographical and ecological information have mostly been invoked descriptively during tree interpretation [29]. One way to integrate these data more objectively is with ecological niche models (ENMs), which use collection localities and Geographic Information System (GIS) maps of environmental data to develop spatial predictions of a species’ historical and current range [29, 30] This technique has to be widely used in Europe and North America phylogeography [30, 31], but rarely used in Qinghai-Tibetan Plateau phylogeography.

*Swertia tetraptera*, belonging to the genus *Swertia* in the Gentianaceae, is an annual herb endemic to the QTP. The main distribution of *S. tetraptera* is in Qinghai, Gansu, and Sichuan Provinces, occurring primarily in moist hillsides and shrub locations with an elevation of 2,000–5,000 m. The main characteristic that distinguishes *S. tetraptera* from other *Swertia* plants is its heteromorphic flowers, that is, every plant has two kinds of flowers: normal ‘open-pollinated’ flowers and ‘closed’ or cleistogamous flowers. As an endemic species of the QTP, *S. tetraptera* formed within its strong uplift (approximately 3.97 Ma) [32]. Therefore, this species is an indispensable part of the study of the influence of the QTP uplift on the distribution pattern of modern plants. To date, only a few studies of *S. tetraptera* based on molecular biology have been reported [33, 34]. Yang et al. (2011) [32] clarified the phylogeography of *S. tetraptera* based on only one chloroplast DNA (cpDNA) fragment. However, this previous study did not concentrate on phylogeographic structure or deep evolutionary history.

In the current study, 301 individuals from 35 populations of *S. tetraptera* were collected from the entire geographic distribution in the high-altitude QTP and adjacent areas. To characterize the population histories of this species, two chloroplast DNA (cpDNA) markers were used to detect genetic variations. The aims were (1) to explore the genetic structure and diversity level of *S. tetraptera* in the Qinghai-Tibetan Plateau; (2) to identify the evolutionary history of *S. tetraptera*; and (3) to infer on the reasons for the existing geographic distribution patterns of *S. tetraptera*.

## Results

### cpDNA variations and haplotype distributions

Two chloroplast gene fragments (*trn*L-*trn*F and *trn*S-*trn*G) were applied to analyze 301 individuals from 35 populations of *S. tetraptera*. The total length of the fragments was 1215 bp, and the lengths of the *trn*L-*trn*F and *trn*S-*trn*G regions were 761 and 454 bp, respectively, which included 22 mutation sites (Supplementary Table S1). Due to the uniparental inheritance of cpDNA, the two chloroplast gene fragments were combined in the subsequent population genetic analysis.

**Fig. 1:**
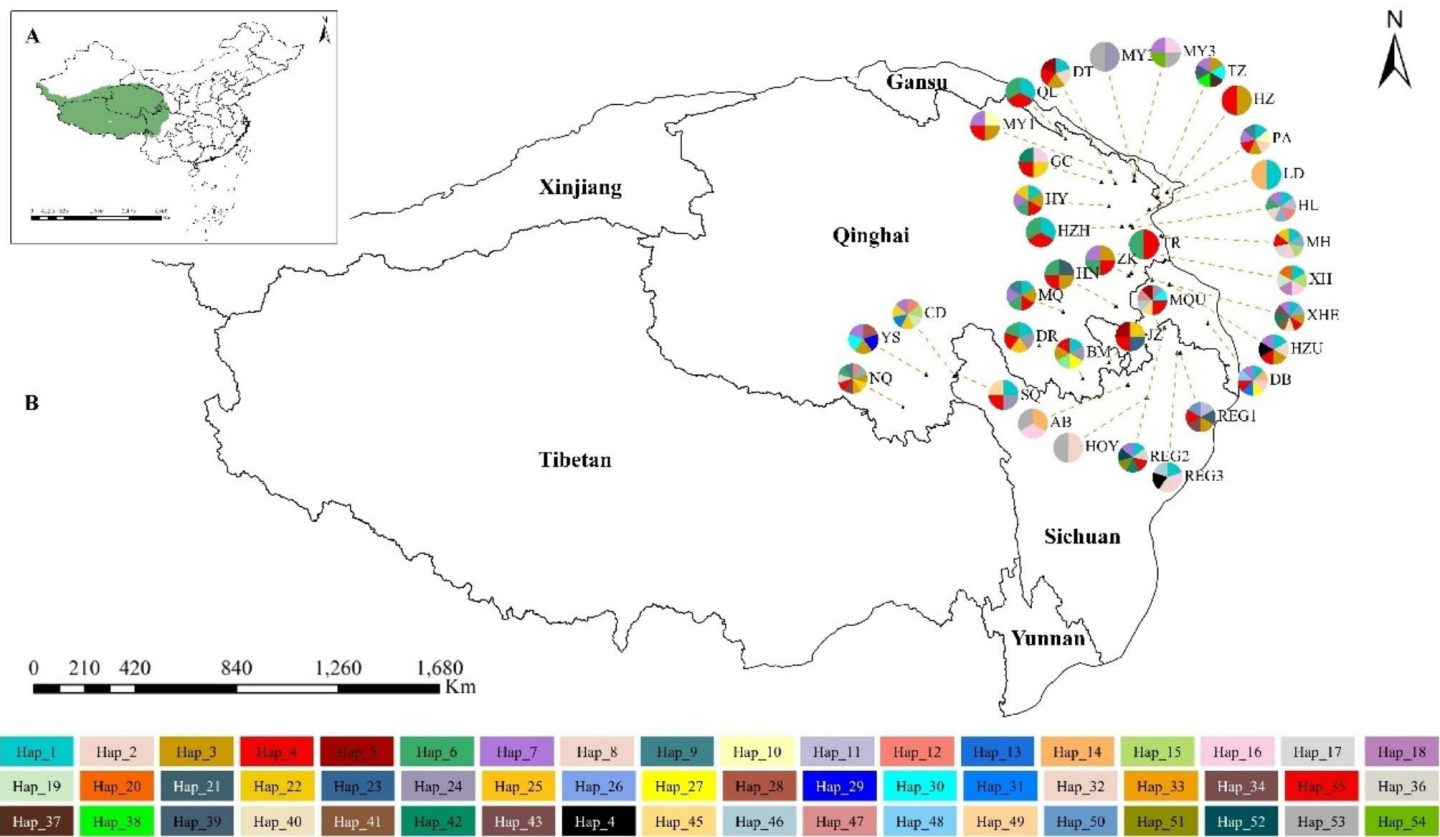
A, map of China, indicating the Qinghai-Tibetan Plateau (Green) and the distribution range of *Swertia tetraptera*. B, map of the 35 sampled populations of *Swertia tetraptera* and the distribution of cpDNA haplotypes in the species. Pie charts show the proportions of haplotypes within each population. The codes of populations are the same as in Table 1. (Note: The background map is taken from an open-access dataset, Integration dataset of Tibet Plateau boundary. National Tibetan Plateau Data Center. Zhang, Y. (2019). Integration dataset of Tibet Plateau boundary. National Tibetan Plateau Data Center. 10.11888/Geogra.tpdc.270099. https://cstr.cn/18406.11.Geogra.tpdc.270099.)

The distribution of the observed haplotypes in each population is indicated in Table 1; Fig. 1. In total, 54 haplotypes were detected in *S. tetraptera*. The main feature of the 54-haplotype distribution was the absence of clear geographic structuring. The most common haplotype, Hap_4, was found in 24 of the 35 populations and made up 23.92% of the total sample. Haplotypes, such as Hap_1, Hap_3, Hap_6, Hap_7, Hap_13 and Hap_53, were also common and were present in 12.96%, 8.64%, 5.32%, 6.31%, 4.32% and 3.32% of the individuals, respectively (Fig. 1; Table 1). The remaining haplotypes were divided into two classes: (i) rare but widely distributed (Hap_5, Hap_8, Hap_9, Hap_10, Hap_11, Hap_12, Hap_13, Hap_14, Hap_15, Hap_19, Hap_21, Hap_22, Hap_24, Hap_26, Hap_29, Hap_30, Hap_31, Hap_32, Hap_34, Hap_35, Hap_36, Hap_38, Hap_42, Hap_44 and Hap_54) and (ii) population-specific (Hap_17, Hap_18, Hap_20, Hap_23, Hap_25, Hap_27, Hap_28, Hap_33, Hap_37, Hap_39, Hap_40, Hap_41, Hap_43, Hap_45, Hap_47, Hap_48, Hap_49, Hap_50, Hap_51 and Hap_52).

**Table 1:** Geographic distributions, gene diversity, nucleotide diversity, and haplotype frequencies of cpDNA sequences for *Swertia tetraptera*

	Population code	Longitude	Latitude	Sample number	Hd	Pi	Haplotype (no. of individuals)
Qilian, Qinghai	QL	100.27ºE	38.16ºN	8	0.679	0.00068	Hap_1(4), Hap_4(3), Hap_6(1)
Mengyuanqingshizui, Qinghai	MY2	101.41ºE	37.46ºN	7	0.810	0.00134	Hap_3(2), Hap_4(3), Hap_7(1), Hap_10(1)
Mengyuanxianmi, Qinghai	MY2	102.01ºE	37.28ºN	4	0.500	0.00083	Hap_24(1), Hap_54(3)
Mengyuanxianmi, Qinghai	MY3	102.02ºE	37.38ºN	11	0.800	0.00321	Hap_7(2), Hap_16(3), Hap_53(4), Hap_54(2)
Huzhu, Qingahi	HZ	102.85 ºE	37.03ºN	11	0.181	0.00015	Hap_3(1), Hap_4(10)
Gangcha, Qinghai	GC	101.18ºE	37.25ºN	7	0.714	0.00291	Hap_4(4), Hap_16(1), Hap_32(1), Hap_42(1)
Datong, Qinghai	DT	101.53ºE	37.22ºN	9	0.806	0.00101	Hap_1(2), Hap_2(1), Hap_3 (1), Hap_4 (4), Hap_5(1)
Huangyuan, Qinghai	HY	101.37ºE	36.73ºN	10	0.867	0.00106	Hap_1(1), Hap_3 (1), Hap_4 (3), Hap_6(3), Hap_7(1), Hap_8(1)
Huangzhong, Qinghai	HZH	101.70ºE	36.30ºN	7	0.667	0.00087	Hap_1(4), Hap_4 (1), Hap_6(2)
Pingan, Qinghai	PA	101.91ºE	36.32ºN	12	0.864	0.00170	Hap_1(1),Hap_2 (1),Hap_3 (4),Hap_4(3),Hap_7(1),Hap_9(1),Hap_10(1)
Hualong, Qinghai	HL	101.97ºE	36.27ºN	11	0.873	0.00183	Hap_1(4),Hap_2(2),Hap_6(1),Hap_7(1),Hap_11(1),Hap_12(1),Hap_13(1)
Ledu, Qinghai	LD	102.39ºE	36.66ºN	8	0.250	0.00083	Hap_1(7), Hap_14(1)
Minghe, Qinghai	MH	102.69ºE	36.09ºN	9	0.944	0.00381	Hap_1(1),Hap_4(4),Hap_8(1),Hap_13(2),Hap_15(1),Hap_16(2),**Hap_17(1)**
Xunhua, Qinghai	XH	102.31ºE	35.74ºN	10	0.778	0.00184	Hap_1(1),Hap_15(5),Hap_16(1),**Hap_18(1)**,Hap_19(1),**Hap_20(1)**
Tongre, Qinghai	TR	101.94ºE	35.31ºN	4	0.500	0.00041	Hap_4(1),Hap_6(3)
Zeku, Qinghai	ZK	101.87ºE	35.25ºN	9	0.694	0.00073	Hap_3(1),Hap_4(1),Hap_6(2),Hap_7(5)
Henan, Qinghai	HN	101.55ºE	34.60ºN	6	0.800	0.00138	Hap_3(1),Hap_4(3),Hap_6(1),Hap_21(1)
Maqing, Qinghai	MQ	100.21ºE	34.47ºN	9	0.833	0.00128	Hap_1(1),Hap_3(1),Hap_4(4),Hap_6(1),Hap_7(1),Hap_9(1)
Jiuzhi, Qinghai	JZ	101.37ºE	33.39ºN	10	0.733	0.00202	Hap_4(5),Hap_5(2),Hap_22(2),**Hap_23(1)**
Dari, Qinghai	DR	99.60ºE	33.75ºN	10	0.667	0.00066	Hap_1(1),Hap_4(6),Hap_6(1),Hap_24(1),**Hap_25(1)**
Banma, Qinghai	BM	100.71ºE	33.05ºN	10	0.889	0.00229	Hap_1(2),Hap_3(3),Hap_4(2),Hap_24(1),Hap_26(1),**Hap_27(1)**
Yushu, Qinghai	YS	96.73ºE	33.13ºN	7	0.905	0.00173	Hap_3(2),Hap_7(1),**Hap_28(1)**,Hap_29(2),Hap_30(1)
Chenduo, Qinghai	CD	97.44ºE	33.11ºN	8	0.964	0.00357	Hap_7(1),Hap_12(1),Hap_15(1),Hap_19(1),Hap_22(1),Hap_31(1),Hap_32(2)
Nangqian, Qinghai	NQ	96.14ºE	32.44ºN	11	0.982	0.00316	Hap_3(1),Hap_6(1),Hap_9(1),Hap_12(1),Hap_13(1),Hap_32(1),**Hap_33(1)**, Hap_34(2),Hap_35(1),Hap_36(1)
Tianzhu, Gansu	TZ	102.60ºE	36.94ºN	8	0.929	0.00177	Hap_3(2),Hap_7(1),Hap_30(1),**Hap_37(1)**,Hap_38(2),**Hap_39(1)**
Xiahe, Gansu	XHE	102.47ºE	35.15ºN	11	0.964	0.00201	Hap_1(2),Hap_3(1),Hap_4(2),Hap_7(1),Hap_13(1),**Hap_40(1)**,**Hap_41(1)**, Hap_42(1),**Hap_43(1)**
Hezuo, Gansu	HZU	102.91ºE	35.06ºN	9	0.917	0.00142	Hap_1(2),Hap_2(1),Hap_3 (2),Hap_4(2),Hap_7(1),Hap_44(1)
Maqu, Gansu	MQU	102.64ºE	34.10ºN	10	0.956	0.00209	Hap_4(2),Hap_5(1),Hap_24(1),Hap_30(1),Hap_35(1),**Hap_45(1)**,Hap_46(2), **Hap_47(1)**
Diebu, Gansu	DB	103.89ºE	34.23ºN	9	0.972	0.00362	Hap_1(1),Hap_4(1),Hap_7(1),Hap_14(1),Hap_16(2),Hap_26(1),Hap_31(1), **Hap_48(1)**
Shiqu, Sichuan	SQ	97.52ºE	33.15ºN	9	0.694	0.00092	Hap_1(2),Hap_4(5),Hap_24(1),**Hap_49(1)**
Ruoergaijiangzha, Sichuan	REG1	103.18ºE	33.60ºN	10	0.867	0.00198	Hap_3(3),Hap_4(3),Hap_11(1),Hap_21(1),Hap_34(1),**Hap_50(1)**
Ruoergaibasi, Sichuan	REG2	102.78ºE	34.14ºN	9	0.917	0.00229	Hap_1(1),Hap_4(3),Hap_7(1),Hap_36(1),Hap_42(1),**Hap_51(1)**,**Hap_52(1)**
Ruoergaibanyou, Sichuan	REG3	103.10ºE	33.59ºN	9	0.861	0.00335	Hap_1(2),Hap_2(2),Hap_16(3),Hap_44(1),Hap_46(1)
Aba, Sichuan	AB	101.85ºE	32.92ºN	5	0.700	0.00149	Hap_14(1),Hap_16(1),Hap_53(3)
Hongyuan, Sichuan	HOY	102.33ºE	32.65ºN	4	0.500	0.00083	Hap_24(1),Hap_54(3)
Total				301	0.908	0.00257	

### Genetic diversity and structure

Unbiased haplotype diversity (*H*d) within the 35 populations ranged from 0.182 to 0.982, and nucleotide diversity (π) ranged from 0.00015 to 0.00362 (Table 1). The NQ (Nangqian, Qinghai) population had the highest haplotype diversity (0.981), and the DB (Diebu, Gansu) population had the highest nucleotide diversity (0.00362). The total gene diversity (*H*_T_) was estimated to be 0.912, and the average gene diversity within populations (*H*_S_) was 0.768. The values of *N*_ST_ and *G*_ST_ were 0.158 and 0.315, respectively. No phylogeographic signal in the haplotype distribution was detected by means of a standard phylogeographic analysis because *N*_ST_ was non-significantly larger than *G*_ST_. AMOVA suggested that most of the genetic variation was found within populations (70.51%) as opposed to between populations (29.49%) (Table 2).

**Table 2:** Results of analysis of molecular variance (AMOVA) of cpDNA sequence data from populations of *S.tetraptera*

Source of variance	d.f.	Sum of squares	Variance components	Percentage total (%)	Fixation index
Among populations	34	172.445	0.46208	29.49	*F*_ST_=0.29491^*^
Within populations	266	293.868	1.10477	70.51	
Total	300	466.312	3.80569		

Note: Vaule marked by an asterisk(^*^) wsa statistically significant at the P < 0.05 level

### Phylogenetic relationships

**Fig. 2:**
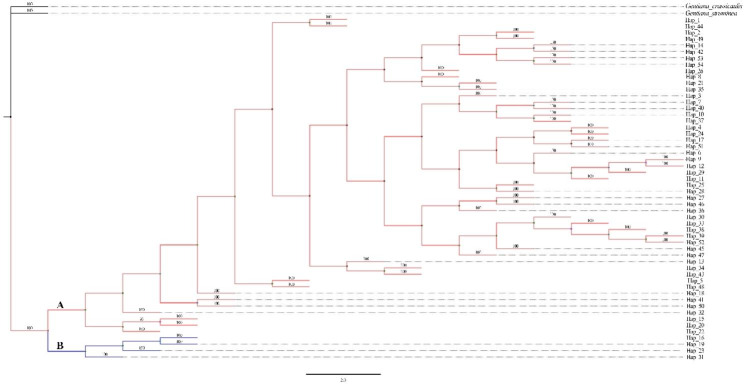
Phylogenetic tree for 54 cpDNA haplotypes using Bayesian inference (BI) method. Posterior probabilities are shown above the branches. A and B represent two different branches

**Fig. 3:**
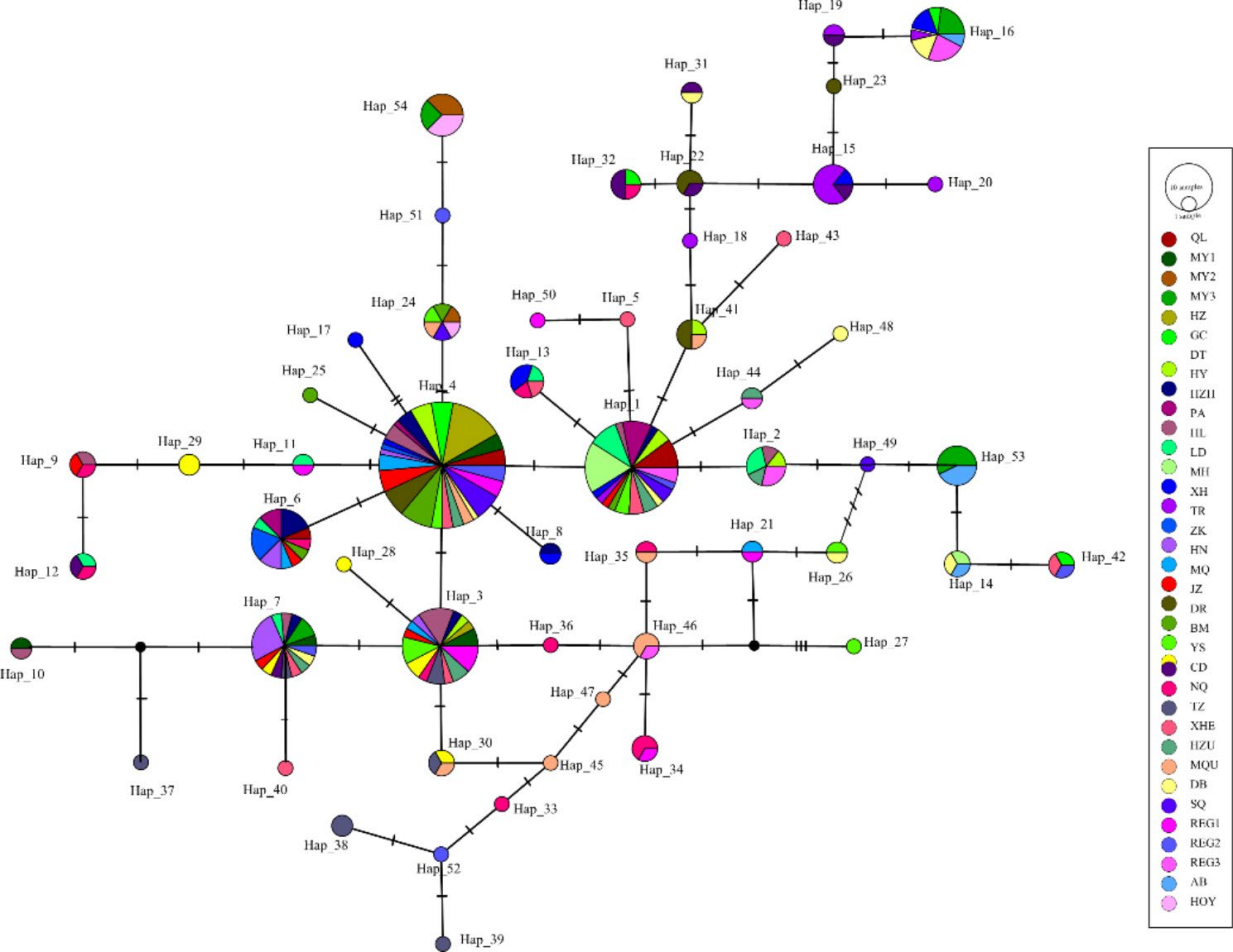
MP median-joining network of the 54 cpDNA haplotypes. Circle size is proportional to haplotype frequencies and the red dot represent missing haplotypes. Each color denotes one population of *S. tetraptera*. The numbers on the branches indicate the number of steps separating adjacent haplotypes

The maximum likelihood and Bayesian methods were used to reconstruct phylogenetic trees for the 54 cpDNA haplotypes in *S. tetraptera*. There was a little difference in the topological structures of the phylogenetic trees obtained by the two methods (Fig. 2, Figure S1). Phylogenetic tree obtained from BI method showed that all 54 cpDNA haplotypes in *S. tetraptera* formed two clades (A and B) (Fig. 2), while phylogenetic tree obtained from ML method demonstrated that all 54 cpDNA haplotypes in *S. tetraptera* formed three clades (A, B and C) (Figure S1). However, haplotypes in different populations mixed in both phylogenetic trees and did not cluster in separate branches according to population. That is, haplotypes lack a distinct geographic distribution structure and disperse into different clades (Figs. 2 and 3). Then, these shallow divergent cpDNA haplotypes were subjected to a MJ network. In the cpDNA network, haplotypes with high distribution frequency (e.g., Hap_4, Hap_1, and Hap_3) were located in the central positions of individual networks, while population-specific haplotypes with low frequency generally occupied network tips (Fig. 3). Divergence between adjacent cpDNA haplotypes was even shallower and was usually distinguished by one mutation step (Fig. 3).

### Population dynamics history and divergence time

**Fig. 4:**
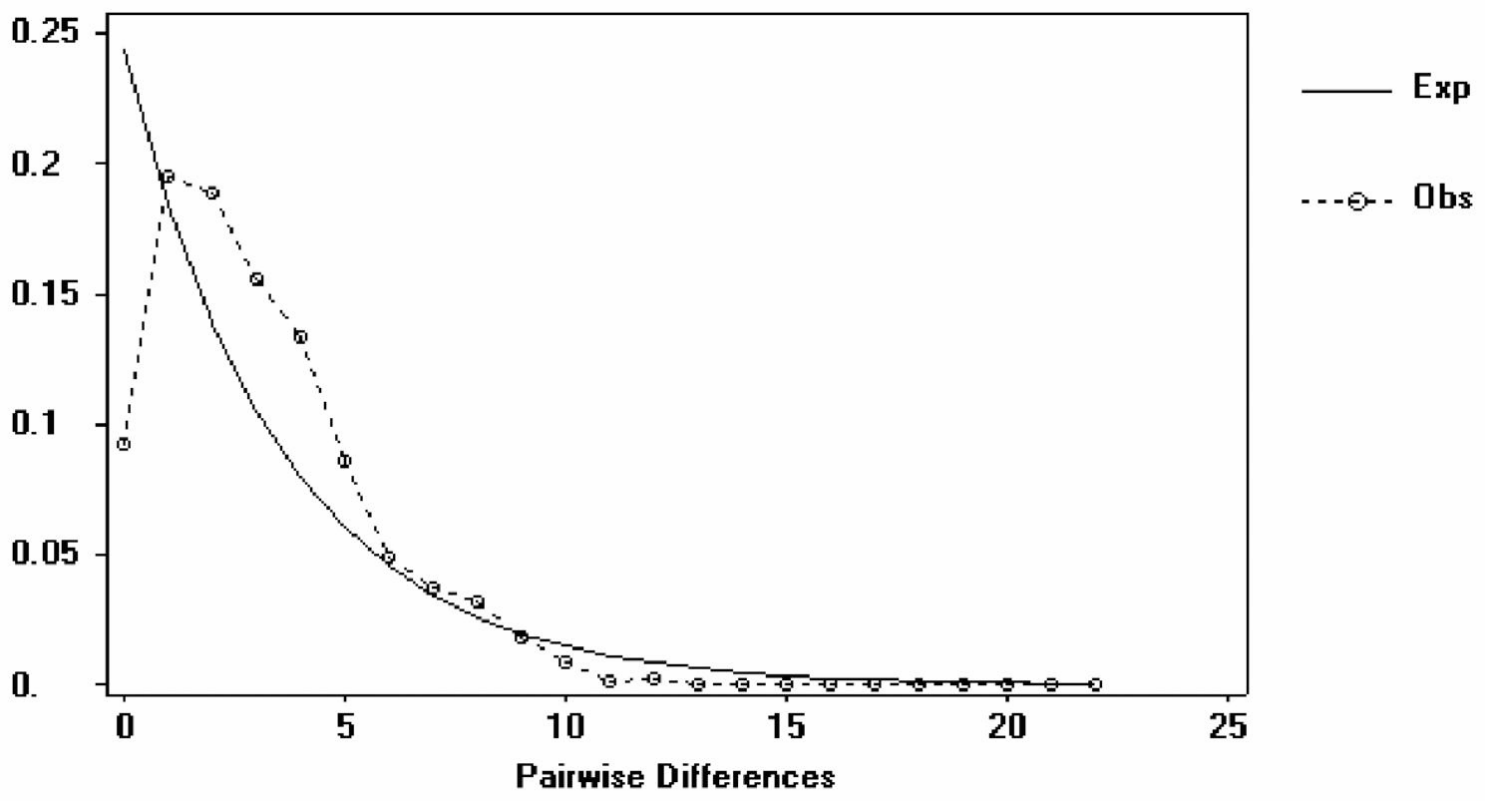
Mismatch distribution of *Swertia tetraptera* in the overall populations based on cpDNA dataset

DnaSP software was used to test the neutrality of all populations based on the chloroplast data of *S. tetraptera*. The results showed that Tajima’s D was − 0.12975 (*P* < 0.001) and Fu’s Fs was − 1.28549 (*P* < 0.001); that is, all were significantly negative values, and the observed mismatch distribution analysis results were single-peak curves (Fig. 4). The results of mismatch distribution analysis were verified by Arlequin software, and the SSD and HRI values were positive and non-significant (SSD = 0.04703, *P* > 0.05; HRI = 0.004, *P* > 0.05). The results indicated that the distribution area or quantity of *S. tetraptera* in its current distribution area had experienced a recent expansion.

### Species distribution modeling

**Fig. 5:**
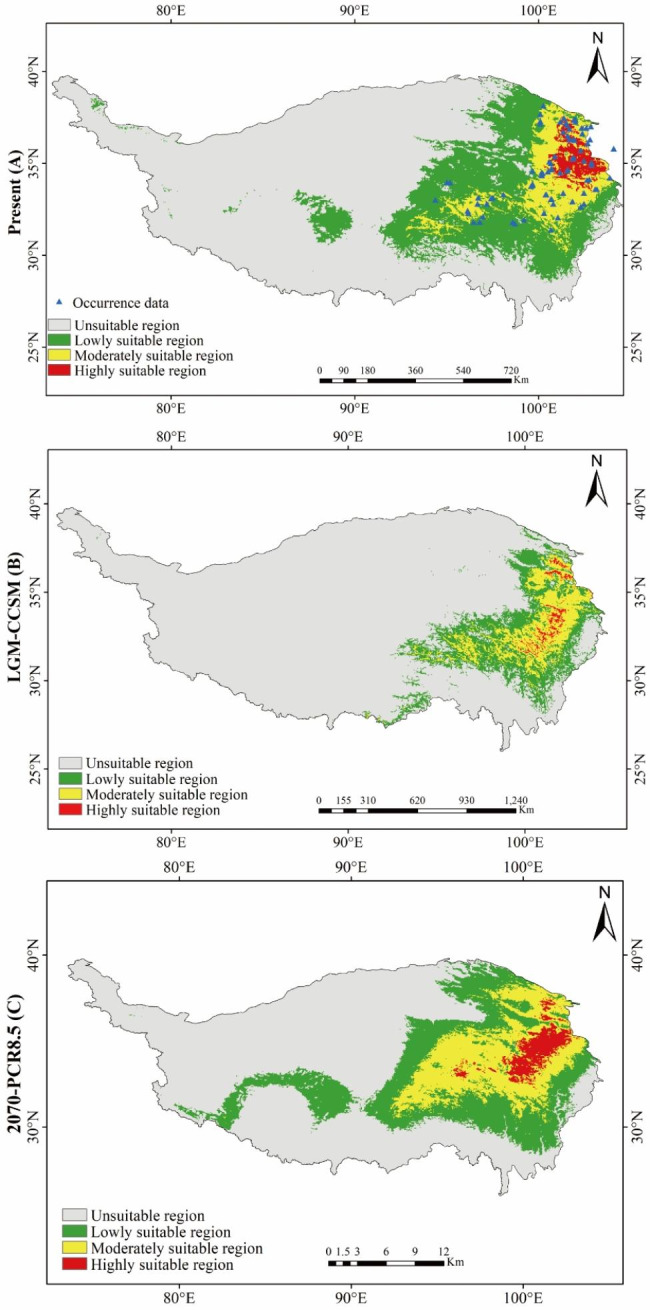
Predicted distribution of *S. tetraptera* based on Present (**A**), LGM (**B**) and Future (**C**) bioclimatic data. The highest probability of occurrence was shown in red color, while the lowest probability of occurrence was shown in green color. (Note: The background map is taken from an open-access dataset, Integration dataset of Tibet Plateau boundary. National Tibetan Plateau Data Center. Zhang, Y. (2019). Integration dataset of Tibet Plateau boundary. National Tibetan Plateau Data Center. 10.11888/Geogra.tpdc.270099. https://cstr.cn/18406.11.Geogra.tpdc.270099.)

**Fig. 6:**
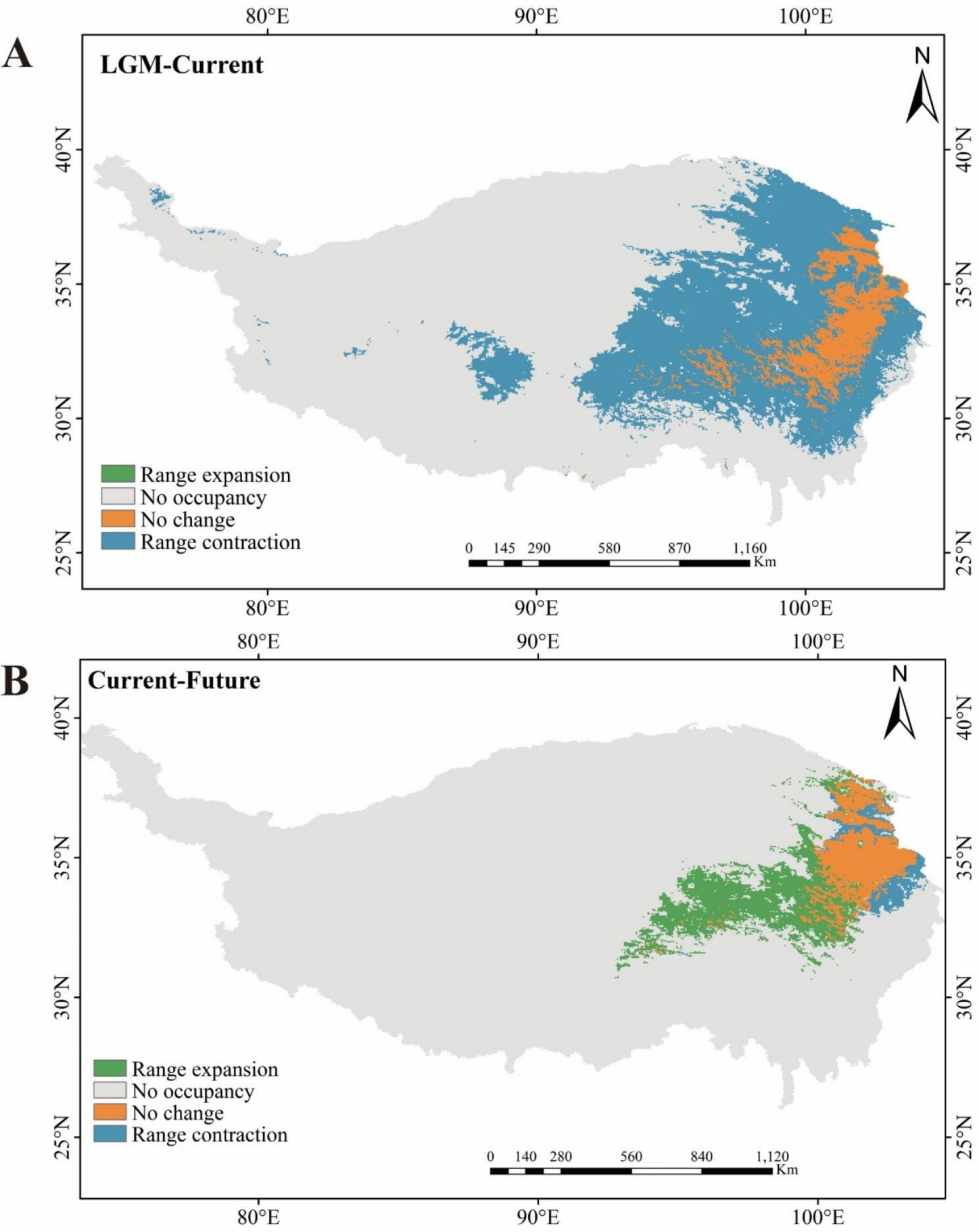
Changes in the distribution of suitable habitats of *S. tetraptera*. The green area indicates that the distribution area of *S. tetraptera* expands in the later period compared with the previous period; the blue area indicates that the distribution area shrinks compared with the previous period; the gray area indicates that there is no distribution in both periods; and the yellow area indicates that there is potential distribution of *S. tetraptera* in both periods. (Note: The background map is taken from an open-access dataset, Integration dataset of Tibet Plateau boundary. National Tibetan Plateau Data Center. Zhang, Y. (2019). Integration dataset of Tibet Plateau boundary. National Tibetan Plateau Data Center. 10.11888/Geogra.tpdc.270099. https://cstr.cn/18406.11.Geogra.tpdc.270099.)

MaxEnt predicted the distributions of *S. tetraptera* during three time periods, LGM (LGM-CCSM4, ~ 21Kya), current (1960–1990), and future (2070s-RCP8.5, 2061–2080), based on selected predictor variables (Fig. 5). The AUC value of the test data was 0.987 (Figure S2), which showed that the prediction result of the model for the potential suitable areas of *S. tetraptera* were very good, and the reliability was relatively high. The environmental variables used to fit the models in *S. tetraptera* were BIO02(Mean Diurnal Range), BIO03(Isothermally (BIO02/BIO07) (100)), BIO04(Temperature seasonality (Standard deviation×100)), BIO10(Mean Temperature of Warmest Quarter), BIO11(Mean Temperature of Coldest Quarter), BIO14 (Precipitation of Driest Month), BIO15(Precipitation Seasonality), BIO18 (Precipitation of Warmest Quarter) (Table S2). The model for the present scenario properly predicted the known distribution range of *S. tetraptera*, assigning high probabilities of occurrence mainly to the Qinghai and Gansu, and dispersed distribution in northwest Sichuan and northeast Xizang (Fig. 6A). The LGM model estimated a shift of the distribution range towards the southern of Qinghai-Tibetan Plateau in response to LGM climates (Fig. 6B). The future scenarios for the distribution of *S. tetraptera* showed a general pattern of expansion of its distribution range when compared to the present (Fig. 6C, Table S3).

### Changes in the distribution of suitable habitats and refuge speculation

In this study, all models built for comparison were tested for regularized multipliers under different conditions (from 0.1 to 2.0), and the ones with good models were finally selected as predictions (AUC = 0.980, RM = 0.1, FC = LQ). Taking the fitness index value 0.0612 as the threshold value for the existence and distribution of *S. tetraptera* in each period, the potential distribution changes of *S. tetraptera* from LGM to current and from current to future were calculated (Fig. 5). The green area indicates that the distribution area of *S. tetraptera* expands in the later period compared with the previous period; the blue area indicates that the distribution area shrinks compared with the previous period; the gray area indicates that there is no distribution in both periods; and the yellow area indicates that there is potential distribution of *S. tetraptera* in both periods.

From LGM to current, the distribution area of *S. tetraptera* in eastern and central of Qinghai-Tibetan Plateau has contracted, while the distribution area in few regions of southern Qinghai-Tibetan Plateau has expanded (Fig. 5A; Table 3). From current to future, its distribution area has expanded in platform of Qinghai-Tibetan Plateau (Fig. 5B; Table 3), while its distribution area has shank in eastern edge of Qinghai-Tibetan Plateau. As can be seen from Fig. 5A and B, the distribution of *S. tetraptera* overlapped in some platform, eastern edge and southeast of Qinghai-Tibetan Plateau before and after LGM. Therefore, these areas may be the potential refuges of *S. tetraptera* during the LGM.

**Table 3:** Distribution changes between time periods

Distribution changes	LGM-Current		Current-Future	
	Area (Km^2^)	Distribution Changes (%)	Area (Km^2^)	Distribution Changes (%)
Range expansion	282.93	+ 0.01	249211.16	+ 9.80
No occupancy	1728504.02	67.97	2092865.13	82.30
No change	131039.94	5.15	101718.84	4.00
Range contraction	683144.42	-26.86	99175.87	-3.90

## Discussion

### Genetic diversity of ***S. tetraptera***

In this study, based on the combined chloroplast fragment, we found that at the species level, the *H*d (haplotype diversity) and π (nucleotide diversity) of *S. tetraptera* were 0.908 and 0.0257, respectively. According to statistics, these values were higher than those of other herbaceous plants on the QTP, such as *Rhodiola chrysanthemifolia* (*H*d = 0.411, π = 0.0025) [35], *Notopterygium incisum* (*H*d = 0.75, π = 0.00086) [22], and *Meconopsis integrifolia* (*H*d = 0.8064, π = 0.000144) [36]. Although the different studies used different molecular markers and the environment, biological characteristics and evolutionary history of the different species are not completely the same, and the biological significance of such indirect comparisons still needs further study, such comparisons can intuitively indicate the degree of genetic diversity of *S. tetraptera*. The high level of genetic diversity in *S. tetraptera* may have been caused by the following. First, there is a strong correlation between plant genetic diversity and the geographical distribution of species [37–39]. Species with a wide natural range usually contain more genetic diversity. Although *S. tetraptera* is an annual herb, it has a wide distribution range and is widely distributed in Qinghai, Gansu, Sichuan and the Tibetan Plateau, China. It can thus be predicted that *S. tetraptera* should have a high level of genetic diversity. This is consistent with the genetic diversity parameters obtained from our analysis of 35 populations. Second, the proportion of endemic haplotypes was high in the populations of *S. tetraptera*, which may have correspondingly increased the level of diversity within populations. Third, the high genetic diversity of *S. tetraptera* may be related to its evolutionary history. According to a previous study, *Swertia* originated in the early Miocene of the Tertiary (29.60 Ma), and the differentiation of *S. tetraptera* was completed in the late Pliocene of the Tertiary (3.97 Ma) (data are presented in the Supplementary Figure S3). Therefore, prior to the Quaternary ice age, *S. tetraptera* may have been widely distributed in the QTP, which may be one of the reasons for its high genetic diversity. In addition, the uplift of the QTP, Himalayan, Hengduan, and Qinling mountains and the inland dry period during the mid-Tertiary led to the differentiation of plants along the direction of adaptation to alpine dry conditions. During the evolution process, *S. tetraptera* experienced different climatic changes and accumulated more genetic diversity under different environmental conditions for its survival by adapting to various environmental pressures.

### Genetic structure of ***S. tetraptera***

In general, the chloroplast data (*G*_ST_=0.315, *F*_ST_=0.2949) indicated a low degree of differentiation among populations of *S. tetraptera*. This may be caused by sufficiently strong gene flow between populations [40, 41]. However, a large number of endemic haplotypes were detected in *S. tetraptera* and which were endemic to most populations, suggesting that gene flow between populations was limited. Although the mechanisms of pollen and seed dispersal in this species are not well understood, a previous study showed that outcrossing and self-crossing existed simultaneously in *S. tetraptera* [33]. However, for populations that grow in extreme environments, where pollen is scarce, self-pollination is especially important to ensure reproduction, which can be confirmed by the special flowers (normal ‘open-pollinated’ flowers and ‘closed’ or cleistogamous flowers). Therefore, effective gene flow was not responsible for the low level of differentiation between populations in *S. tetraptera*. Thus, rapid intraspecific diversification was one of the reasons for the low degree of differentiation among populations of *S. tetraptera*. Of course, it is possible that other factors, such as hybridization and/or introgression, contributed to the low level of differentiation between populations in *S. tetraptera*, but we do not currently know whether this phenomenon exists in this species. According to our long-term observations in the field, it was found that the distribution of *S. tetraptera* and *Halenia elliptica* was sympatric. When plant spacing between sympatric species is less than 10 cm, geographical opportunities for hybridization and/or gene introgression between them are provided. Therefore, considering the genetic structure of the population and the close relationship between *S. tetraptera* and *H. elliptica*, as indicated in previous studies [42–48], it is inferred that there may be hybridization and/or gene introgression between *S. tetraptera* and its sympatric species *H. elliptica*.

A large number of private haplotypes existed in the current populations of *S. tetraptera*, and a small number of widely distributed haplotypes with high frequency occurred among populations, which might reduce the degree of differentiation between populations to some extent. In addition, limited mutation sites were detected in many haplotypes, and adjacent haplotypes were separated by a limited number of mutation steps. Due to the limited number of mutation sites of haplotypes generated by cpDNA fragments, clear results were not obtained when constructing ML and BI phylogenetic relationship trees. The low mean nucleotide diversity among populations further confirmed that the differentiation of the chloroplast haplotypes of this species was shallow. Therefore, the lack of phylogeographic structure in *S. tetraptera* might be the consequence of the low occurrence frequency, scattered distribution and shallow differentiation of the endemic haplotypes, which was similar to *Saxifraga sinomontana* [49], *Rhodiola chrysanthemifolia* [35], *R. alsia* [16], *Stellera chamaejasme* [50], and *Potentilla glabra* [21].

### Glacial refugia of ***S. tetraptera***

Ice refugium is the concentration of plants to escape from the harsh climate of ice age, and also the starting point of population redistribution after ice age [51]. Compared with redispersal populations, the evolutionary history of plant populations in refugium is able to accumulate rich genetic diversity. However, the dispersal from refugium is often accompanied by genetic drift or founder effect, and haplotype polymorphism or gene polymorphism decreases with the increase of population dispersal distance. Therefore, the level of genetic diversity can be used as an important basis to infer the specific location of shelters [52]. A summarization of the QTP alpine species investigated to date shows that the ‘‘contraction/recolonization’’ hypothesis, ‘‘platform refugia/local expansion’’ hypothesis and ‘‘microrefugia’’ hypothesis are the three main phylogeographic patterns of plant species on the QTP during the Quaternary glaciations [50, 53]. In this study, many private haplotypes were dispersed across the distribution range of *S. tetraptera*, and populations with high genetic diversity demonstrated an even distribution. Moreover, ancestral (Hap_4) and unique haplotypes appeared in the same population (Table 1; Fig. 1). All these results strongly suggest the existence of multiple microrefugia of *S. tetraptera* in the QTP. This result was also supported by the ENM. The distribution of *S. tetraptera* overlapped in the eastern, southeast and southwest Qinghai, southern and north Gansu, and southwest of Sichuan before and after LGM. Therefore, these areas may be the potential refuges of *S. tetraptera* during the LGM. At the LGM, the range of *S. tetraptera* showed a dispersed distribution in the QTP (Fig. 6B). In fact, taking into consideration the topological heterogeneity of the QTP and rejecting the claim that there was no unified ice sheet on the QTP during the ice age [54], it was likely that suitable microenvironments existed for cold-tolerant herbs [35] to survive glaciations in situ. However, both mismatch distribution analysis and neutrality tests showed a recent expansion across the distribution range of *S. tetraptera*. As mentioned above, combining the present genetic structure of *S. tetraptera* with the low dispersal ability of seeds provided evidence of extensive horizontal range expansion across the distribution range of *S. tetraptera*. Therefore, the discovered expansion signal possibly represents demographic expansion or altitudinal migration in response to repeated glacier advances and retreats, which was also detected in *R. chrysanthemifolia* [35], *Potentilla fruticosa* [55] and *P. glabra* [21]. As a result, we inferred that *S. tetraptera* had a continuous distribution in the QTP region before the Quaternary glaciations and had some widespread haplotypes (such as Hap_4 and Hap_3) across its distribution range. After the repeated advance and retreat of the glaciers during the Quaternary, the distribution range of *S. tetraptera* may have fragmented into isolated patches, ultimately facilitating in situ allopatric divergence. Since then, affected by bottleneck effects caused by Quaternary glaciations, the number of individuals in *S. tetraptera* population decreased dramatically, and only a few individuals remained, the new population developed from these individuals retained only part of the ancestral population genotype (Hap_4 and Hap_3). Finally, *S. tetraptera* has lost some of the ancient genetic structure to some degree, producing a large number of unique haplotypes, which eventually formed the existing genetic structure.

In this study, only the effects of climate change on the distribution range, population structure and population dynamics of *S. tetraptera* were analyzed, while the natural geographical barriers such as mountains and rivers and human activities could also affect the evolution and dispersal of *S. tetraptera*, which were not involved in this study. Therefore, we should continue to study the differentiation pathway and evolutionary mechanism of *S. tetraptera* in the future, so as to provide a more comprehensive and richer theoretical basis for the utilization and protection of this species.

## Conclusion

Our study indicates that the current geographic and genetic distribution of *S. tetraptera* is likely to have been shaped by Quaternary periods. Multiple microrefugia of *S. tetraptera* existed during Quaternary glaciations. Rapid intraspecific diversification and hybridization and/or introgression may have played a vital role in shaping the current distribution patterns of *S. tetraptera.* The distribution range of *S. tetraptera* appeared to have experienced contraction during the LGM; in the future, when the global climate becomes warmer with rising carbon dioxide, the distribution of *S. tetraptera* will expand.

## Materials and methods

### Population sampling

During the summers of 2008 and 2009, we collected samples throughout the range of *S. tetraptera*. Fresh leaves were collected from 35 populations, and with few exceptions, 4–12 individuals that were at least 50 m apart from each other were sampled from each population (Table 1; Fig. 1). We measured the longitude, latitude and altitude of each collection location using an Etrex Global Positioning System device (Garmin). In total, 301 individuals were sampled, and leaves were dried with silica gel.

### DNA extraction, amplification and sequencing

Total genomic DNA was extracted from silica gel-dried leaves using the modified CTAB method [56] and used as a template in the polymerase chain reaction (PCR). Preliminary universal primer scans of chloroplast DNA genomes were performed on 10 individuals from 10 different populations using 5 pairs of primers. Primers a and f of Taberlet et al. (1991) [57] were used to amplify the *trn*T-*trn*F region and sequenced with primers **a**, **c** and **f**. The other four regions, *psb*B-*psb*H, *rpl*20–5′*rps*12, *trn*S-*trn*G and *psb*A-*trn*H, were amplified and sequenced using primers described in Hamilton (1999) [58]. The primers used to amplify *trn*S-*trn*G showed different sequences within the 10 individuals tested and were used thereafter for the large-scale survey of haplotype variation within *S. tetraptera*. PCR was performed in a 25 µL volume containing 10–40 ng plant DNA, 50 mM Tris-HCl, 1.5 mM MgCl_2_, 250 µg/mL BSA, 0.5 mM dNTPs, 2 µM of each primer, and 1 unit of *Taq* polymerase. Initial template denaturation was programmed at 95 °C for 5 min, followed by 35 cycles of 94 °C for 1 min, 52 °C for 1 min, and 72 °C for 1 min plus a final extension of 72 °C for 7 min. The BioDev Gel Extraction System B Kit (BioDev-Tech) was used to purify all successfully amplified DNA fragments. The PCR primers *trn*S and *trn*G were adopted to perform sequencing reactions using the ABI Prism Bigdye™ Terminator Cycle Sequencing Ready Reaction Kit. All *trn*L-*trn*F sequences of *S. tetraptera* used in this study were obtained from Yang et al. (2011) [33].

### Proofreading and alignment of DNA and data analysis

We used BioEdit v 7.0.9.0 [59] software for manual proofreading and examining the variable sites. Then, we used MEGA v 7.0 [60] software to eliminate low-quality sequences, and we only used high-quality sequences for subsequent analysis. Sequence Matrix 1.8 was used to combine the *trn*L-*trn*F and *trn*S-*trn*G gene segments, and the combined cpDNA gene segment was used to carry out the subsequent analysis.

### Genetic variation and genetic structure analysis

We used the program ARLEQUIN version 3.5.2 [61] to calculate the unbiased genetic diversity (*H*d) and nucleotide diversity (*π*) indices for each population. Differentiations among populations and within populations were calculated by analysis of molecular variance (AMOVA) using ARLEQUIN version 3.5.2.

The program PERMUT was used to calculate the total gene diversity (*H*_T_), average gene diversity within populations (*H*_S_), coefficient of genetic variation over all populations (*G*_ST_) and equivalent coefficient taking into account sequence similarities between haplotypes (*N*_ST_) [62]. A comparison was made between *G*_ST_ and *N*_ST_ using a permutation test with 1000 permutations. The phylogeographic structure appears when the *N*_ST_ value is significantly larger than the *G*_ST_ value.

### Phylogenetic analysis

We used DnaSP v5.10 to identify haplotypes of the cpDNA genes. Haplotype distribution was visualized by GenGIS [63]. The program Popart 1.7 [64] (available at http://popart.otago.ac.nz/index.shtml) was used to construct the haplotype network based on Median joining network [65].

In this study, a phylogenetic tree was constructed based on Bayesian inference (BI) [66] and maximum likelihood (ML) [67] using *Gentiana straminea* (HM598090 for *trn*S-*trn*G intergenic spacer and HM598120 for *trn*L-*trn*F intergenic spacer) and *Gentiana crassicaulis* (DQ398745 for *trn*S-*trn*G intergenic spacer and KT907776 for *trn*L-*trn*F intergenic spacer) as the outgroup. MAFFT 7.205 software was used to compare the cpDNA sequences and remove the irregular sequences at both ends [68]. Before building the BI tree, PAUP and MrModeltest were jointly run through MrMTgui. The Akaike information criterion (AIC) results showed that the best model for BI analysis was GTR + I + G, with a random tree as the starting tree. Starting with four Markov chains, namely, three hot chains and one cold chain, one tree was saved every 100 generations, and 9,000,000 generations were calculated in total, after which the first 25% preheated (burn-in) trees were discarded, and the remaining trees were used to calculate the Bayesian posterior probability (PP) of the consistent trees and of each branch. PAUP 4.0b10 software was used to construct the ML tree and bootstrap support (BS) was used to evaluate the reliability of each branch of ML phylogenetic tree [69].

### Demographic history

Mismatch distribution and neutral tests for the existing populations of *S. tetraptera* were conducted with DnaSP ver. 5.10 [70] software. If the result of mismatch distribution is unimodal, it indicates that the population may have experienced recent expansion. If it is multiple peaks, it means that the population size is relatively stable and in individual equilibrium for a long time [71]. For the neutral test, two infinite mutation-site model indices, Tajima’s D and Fu’s Fs [72–74], were selected to predict the nature of sequence evolution and possible population history dynamics. Negative values of the two indices indicate that the population may have undergone recent expansion or selective sweep. Positive values of the two indices indicate that populations may have been geographically isolated for a long time and that mutation differences between populations were accumulated or controlled by balanced selection [70, 74]. Arlequin ver. 3.5.2 [61] was used to test the results of mismatch distribution analysis, among which sum of square deviation (SSD) and Harpending’s raggdeness index (HRI) were used to test whether to accept the hypothesis of recent rapid population expansion.

### Species distribution modeling

#### Data description

#### Species occurrence data

A total of 97 samples were collected in this study between 1960 and 2020, covering the known distribution areas of *S.tetraptera*. The geographic distribution data of *S.tetraptera* were obtained mainly by the following methods: (1) field investigation (for detailed information, see Table 1); (2) the network data, including the China digital plant herbarium (http://www.cvh.org.cn/), China plants subject database (http://www.plant.csdb.cn/) and Global Biodiversity Information Facility (https://www.gbif.org/). (3) Literature review, including Chinese and English journals, flora of China, flora of local areas, investigation reports of nature reserves, etc. We selected the reasonable sampling sites according to the following principles: First, only one record of the duplicate site data was retained, and the distance between the two sampling sites was more than 10 km. Second, the sampling points must have accurate latitude and longitude information (distribution points that had place name no geographic coordinate information were removed) to ensure the accuracy of geography. Third, to reduce the overfitting of model predictions due to species distribution clusters, spatial auto-correlation analysis was performed using the Perl script-based GUI ENMTools v1.4.4 (http://www.ENMTools.com) and followed the selection and elimination criteria provided by previous researchers [75–78] (http://www.ENMTools.com). Finally, 83 records of species occurrence data were obtained for analysis (Supplementary Table S4).

#### Environmental data

In this study, nineteen bioclimatic variables in the global circulation model of the Community Climate System Model (CCSM4) for the period of 1970‒2000 were obtained from the WorldClim dataset, with a resolution of 30 arcseconds or approximately 1 km^2^ per pixel [79]. To estimate the potential distributions of *S. tetraptera* in the past and the future, the climatic scenarios of the LGM (Last Glacial Maximum, approximately 22,000 years ago, with a resolution of 2.5 arcminutes or approximately 5 km^2^ per pixel); 2070s (average for 2061–2080, with a resolution of 30 arcseconds or approximately 1 km^2^ per pixel), were used for the modelling. For future climate scenarios, we used one future climate projection, CCSM4 from CMIP5 [80] that were downloaded from the World Climate Database version 1.4. Representative concentration pathways (RCPs) for maximum (8.5 W/m^2^) emission hypothesis over the period 2070 were selected for further projections. The RCPs reflect potential radiative forcing by 2100 compared with the pre-industrial values of + 8.5 W/m^2^ which is more pessimistic and reflects high emission levels of greenhouse gases [79]. To facilitate the spatial analysis within Qinghai-Tibetan Plateau, all variables (past, present and future) that were selected for modelling were resampled to 2.5 arcminutes (approximately 5 km^2^) by using the spatial analysis tools in ArcGIS 10.6 (ESRI Inc, California, USA) [81, 82]. The layers were processed using a system with identical projection (WGS84), cell size, and extent, cropped to the region of interest, and converted into .asc format using Spatial Analyst and Conversion tools in ArcGIS 10.5 (ArcGIS, 2016).

### MaxEnt model processing

At present, maximum entropy (MaxEnt) modelling is one of the most popular distribution models [83, 84], which was used to predict the species potential distribution based on current geographic locations and associated environmental variables and provide a spatial representation of habitat suitability on a scale from 0 (lowest suitability) to 1 (highest suitability). We used the MaxEnt model to predict the past, current and future potential distribution of *S. tetraptera* in the Qinghai-Tibetan Plateau. The main factors affecting the MaxEnt modelling results include highly multicollinear bioclimatic variables, regularization parameters and feature classes. Herein, we address this issue in two main ways. First, for the 19 climate variables, the “cor” function in R was used to remove the highly correlated variables and retain with Pearson statistic values < 0.85 [85, 86], which can reduce data redundancy and improve prediction accuracy. Second, we used the Kuenm package (https://github.com/marlonecobos/kuenm) to optimize the regularization multiplier and feature class parameters in the R version 3.6.3 software (Supplementary Model_calibration.PDF). AICc values indicate how well models fit the data while penalizing complexity to favor simple models [38]. Therefore, for *S. tetraptera*, 1160 candidate models were created using the kuenm package to test candidate solutions for each of the environmental variables, all possible combinations of the feature types (linear = l, quadratic = q, product = p, threshold = t, and hinge = h), and regularization multiplier settings (0.1–2.0 at intervals of 0.1) [79, 87]. Finally, using the kuenm and AICc values (the class with the lowest delta AICc was preferred), the best set of candidate solutions that feature combination (FC), including L and Q, and the regularization multiplier, set to 0.1, was chosen [88]. Based on the above optimized parameters, MaxEnt v3.4 was used to build current niche model. The 83-occurrence data of *S. tetraptera* and eight environmental variables are imported into MaxEnt v3.4 [89] for calculation, and the result is logistic output [82, 90–92]. For evaluation of the predictive performance of the model, 75% of the data was used for training, and the remaining 25% was used to evaluate the performance of the model [93–95]. The model was set to be run repeatedly for 10 times, the maximum background point and the number of iterations were set to 10,000 and 5000 respectively, and the rest parameters were set to the default values [88, 96, 97]. The model trained with current climate data was projected on past and future climate data to determine potential distributions in the LGM and 2070s. Average output (based on ten replicates) was used for further analyses. The MaxEnt results were validated using the threshold-independent area under the curve (AUC) of receiver operating characteristics (ROC) [98–100]. Jackknife analyses were performed to assess the importance of the variables [101]. The output of the model is a continuous probabilistic layer, ranging from 0 to 1. Areas with higher values imply more favorable conditions for the species growth [25]. We selected the maximum test sensitivity plus specificity logistic threshold as a threshold or “cutoff” value for each scenario, which is very robust with all types of data [102]. The threshold is equal to 0.0612 for *S. tetraptera*. And then, habitat suitability was divided into four classes based on nature breaks through ArcGIS with 0.0612 as a threshold: unsuitability (0-0.0612), low suitability (0.0612–0.3741), moderate suitability (0.3741–0.6871), and high suitability (0.6871-1).

For the three ENMs, ensemble models were averaged prior to conversion to a binary model. The final ensemble ENMs were converted to a binary model by classifying values greater than (or equal to) 0.5 to a value of 1 and those lower to a value of 0 [103]. This model represented the predicted binary distribution of *S. tetraptera*. To measure the predicted distribution changes for *S. tetraptera*, the binary ENMs were projected to Qinghai-Tibetan Plateau projection in ArcGIS 10.5 using SDMtoolbox v2.2 [104]. The Last Glacial Maximum and Present ENMs, present and future ENMs were then subtracted from each other, and areas of contraction, expansion and stability were calculated, generating a map displaying the intensity of contraction, expansion, and stability throughout its distribution area of the species. Combined with the three periods of suitable distribution overlapping area, the refugium of *S. tetraptera* was inferred [53, 78].

### Electronic supplementary material

Below is the link to the electronic supplementary material.

Supplementary Material 1

Supplementary Material 2

Supplementary Material 3

Supplementary Material 4

Supplementary Material 5

Supplementary Material 6

## Data Availability

All data generated or analyzed during this study are included in this published article and its supplementary information files. The datasets generated and/or analyzed during the current study are available in the GenBank repository (the accession numbers of *trn*L-*trn*F are OQ354846-OQ354860 https://www.ncbi.nlm.nih.gov/nuccore/OQ354846, https://www.ncbi.nlm.nih.gov/nuccore/OQ354860), and the accession numbers of *trn*S-*trn*G are OQ377134-OQ377201, https://www.ncbi.nlm.nih.gov/nuccore/OQ377134, https://www.ncbi.nlm.nih.gov/nuccore/OQ377201).
