# Supplementary material for: Phylogeography and ecological niche modeling implicate multiple microrefugia of Swertia tetraptera during quaternary glaciations
Source: BMC Plant Biol. 2023 Sep 26;23:450. doi: 10.1186/s12870-023-04471-w (PMC10521563; doi:10.1186/s12870-023-04471-w)
Supplement: Supplementary file 1 — Supplementary Material 1 [file 12870_2023_4471_MOESM1_ESM.docx]

Table S1 Variable sites of haplotypes

|  |  |  | | Variable Site | | | | | | | | | | | | | | | | | | | | |
| --- | --- | --- | --- | --- | --- | --- | --- | --- | --- | --- | --- | --- | --- | --- | --- | --- | --- | --- | --- | --- | --- | --- | --- | --- |
| Hap_1 | A | | G | | G | G | G | T | A | G | T | G | A | A | G | A | G | A | G | T | T | T | T | A |
| Hap_2 | **.** | | **.** | | **.** | **.** | **.** | **.** | **.** | **.** | **.** | **.** | **.** | **.** | **.** | G | **.** | **.** | **.** | **.** | **.** | **.** | **.** | **.** |
| Hap_3 | **.** | | **.** | | **.** | **.** | **.** | **.** | **.** | A | **.** | **.** | **.** | **.** | T | **.** | **.** | **.** | **.** | **.** | **.** | **.** | **.** | **.** |
| Hap_4 | **.** | | **.** | | **.** | **.** | **.** | **.** | **.** | A | **.** | **.** | **.** | **.** | **.** | **.** | **.** | **.** | **.** | **.** | **.** | **.** | **.** | **.** |
| Hap_5 | **.** | | **.** | | **.** | **.** | **.** | **.** | **.** | **.** | **.** | **.** | **.** | **.** | **.** | **.** | **.** | G | **.** | **.** | **.** | **.** | **.** | **.** |
| Hap_6 | **.** | | **.** | | **.** | **.** | **.** | **.** | **.** | A | **.** | A | **.** | **.** | **.** | **.** | **.** | **.** | **.** | **.** | **.** | **.** | **.** | **.** |
| Hap_7 | **.** | | **.** | | **.** | **.** | **.** | **.** | **.** | A | **.** | A | **.** | **.** | T | **.** | **.** | **.** | **.** | **.** | **.** | **.** | **.** | **.** |
| Hap_8 | **.** | | **.** | | **.** | **.** | **.** | **.** | **.** | A | **.** | **.** | **.** | **.** | **.** | G | **.** | **.** | **.** | **.** | **.** | **.** | **.** | **.** |
| Hap_9 | **.** | | **.** | | **.** | **.** | **.** | **.** | **.** | A | **.** | A | **.** | **.** | **.** | **.** | **.** | **.** | **.** | **.** | **.** | G | G | . |
| Hap_10 | **.** | | **.** | | **.** | A | **C** | **.** | **.** | A | **.** | A | **.** | **.** | T | **.** | **.** | **.** | A | **.** | **.** | G | **.** | **.** |
| Hap_11 | **.** | | **.** | | **.** | **.** | **.** | **.** | **.** | A | **.** | **.** | **.** | **.** | **.** | **.** | **.** | **.** | **.** | **.** | **.** | G | **.** | **.** |
| Hap_12 | **.** | | **.** | | **.** | **.** | **.** | **.** | **.** | A | **.** | A | **.** | **.** | T | **.** | **.** | **.** | **.** | **.** | **.** | G | G | **.** |
| Hap_13 | **.** | | **.** | | **.** | **.** | **.** | **.** | **.** | **.** | **.** | **.** | **.** | **.** | T | **.** | **.** | **.** | **.** | **.** | **.** | **.** | **.** | **.** |
| Hap_14 | **.** | | **.** | | **.** | **.** | **.** | **.** | **.** | **.** | **.** | **.** | G | **.** | **.** | G | **.** | G | **.** | **.** | **.** | **.** | **.** | G |
| Hap_15 | T | | **.** | | **.** | A | **C** | **.** | - | **.** | **.** | **.** | **.** | **.** | **.** | **.** | A | G | **.** | **.** | **.** | **.** | **.** | G |
| Hap_16 | T | | **.** | | **.** | **.** | **.** | **.** | **-** | **.** | **.** | **.** | G | G | **.** | G | A | G | **.** | **.** | **.** | **.** | **.** | G |
| Hap_17 | **.** | | **.** | | T | **.** | **.** | A | **.** | A | **.** | **.** | **.** | **.** | **.** | **.** | **.** | **.** | **.** | **.** | **.** | **.** | **.** | **.** |
| Hap_18 | **.** | | **.** | | **.** | **.** | **.** | **.** | **-** | **.** | **.** | **.** | **.** | **.** | **.** | **.** | A | G | **.** | **.** | **.** | **.** | **.** | **.** |
| Hap_19 | T | | **.** | | **.** | **.** | **.** | **.** | **-** | **.** | **.** | **.** | G | **.** | **.** | G | A | G | **.** | **.** | **.** | **.** | **.** | G |
| Hap_20 | T | | **.** | | T | **.** | **C** | **.** | - | . | **.** | **.** | **.** | **.** | **.** | **.** | **.** | G | **.** | **.** | **.** | **.** | **.** | G |
| Hap_21 | **.** | | **.** | | **.** | **.** | **.** | **.** | **.** | A | **.** | **.** | **.** | **.** | **.** | G | **.** | G | **.** | **.** | **.** | **.** | **.** | G |
| Hap_22 | T | | **.** | | **.** | **.** | **.** | **.** | **-** | **.** | **.** | **.** | **.** | **.** | **.** | **.** | A | G | **.** | **.** | **.** | **.** | **.** | **.** |
| Hap_23 | T | | **.** | | **.** | **.** | **.** | **.** | **-** | **.** | **.** | **.** | **.** | **.** | **.** | G | A | G | **.** | **.** | **.** | **.** | **.** | G |
| Hap_24 | **.** | | **.** | | **.** | **.** | **.** | **.** | **.** | A | **.** | **.** | **.** | **.** | **.** | **.** | **.** | **.** | **.** | **.** | **.** | **.** | **.** | G |
| Hap_25 | **.** | | A | | **.** | **.** | **.** | **.** | **.** | A | **.** | **.** | **.** | **.** | **.** | **.** | **.** | **.** | **.** | **.** | **.** | **.** | **.** | **.** |
| Hap_26 | **.** | | **.** | | **.** | **.** | **.** | **.** | **.** | **.** | **.** | **.** | **.** | **.** | **.** | G | **.** | G | **.** | **.** | **.** | **.** | **.** | G |
| Hap_27 | **.** | | **.** | | **.** | **.** | **.** | **.** | **.** | A | G |  | **.** | **.** | T | G | **.** | G | **.** | **.** | **.** | G | G | G |
| Hap_28 | **.** | | A | | **.** | **.** | **.** | **.** | **.** | A | **.** | **.** | **.** | **.** | T | **.** | **.** | **.** | **.** | **.** | **.** | **.** | **.** | **.** |
| Hap_29 | **.** | | **.** | | **.** | **.** | **.** | **.** | **.** | A | **.** | A | **.** | **.** | **.** | **.** | **.** | **.** | **.** | **.** | **.** | G | **.** | **.** |
| Hap_30 | **.** | | **.** | | **.** | **.** | **.** | **.** | **.** | A | **.** | **.** |  | **.** | T | **.** | **.** | **.** | **.** | **.** | A | **.** | **.** | **.** |
| Hap_31 | T | | **.** | | **.** | **.** | **.** | **.** | **-** | **.** | **.** | **.** | G | **.** | **.** | **.** | A | G | **.** | **.** | **.** | **.** | **.** | **.** |
| Hap_32 | T | | **.** | | **.** | **.** | **.** | **.** | **-** | **.** | **.** | **.** | **.** | **.** | **.** | **.** | A | **.** | **.** | **.** | **.** | **.** | **.** | **.** |
| Hap_33 | **.** | | **.** | | **.** | **.** | **.** | **.** | **.** | A | **.** | **.** | **.** | **.** | T | G | **.** | **.** | **.** | A | A | **.** | **.** | **.** |
| Hap_34 | **.** | | **.** | | **.** | **.** | **.** | **.** | **.** | **.** | **.** | **.** | **.** | **.** | T | G | **.** | G | **.** | **.** | **.** | **.** | **.** | **.** |
| Hap_35 | **.** | | **.** | | **.** | **.** | **.** | **.** | **.** | A | **.** | **.** | **.** | **.** | **.** | G | **.** | G | **.** | **.** | **.** | **.** | **.** | **.** |
| Hap_36 | **.** | | **.** | | **.** | **.** | **.** | **.** | **.** | A | **.** | **.** | **.** | **.** | T | G | **.** | **.** | **.** | **.** | **.** | **.** | **.** | **.** |
| Hap_37 | **.** | | **.** | | **.** | **.** | **.** | **.** | **.** | A | **.** | A | **.** | **.** | T | **.** | **.** | **.** | A | **.** | A | **.** | **.** | **.** |
| Hap_38 | **.** | | **.** | | **.** | **.** | **.** | **.** | **.** | A | **.** | A | **.** | **.** | T | **.** | **.** | **.** | **.** | A | A | **.** | **.** | **.** |
| Hap_39 | **.** | | **.** | | **.** | **.** | **.** | **.** | **.** | A | **.** | **.** | **.** | **.** | **.** | **.** | **.** | **.** | **.** | A | A | **.** | **.** | **.** |
| Hap_40 | T | | **.** | | **.** | **.** | **.** | **.** | **.** | A | **.** | A | **.** | **.** | T | **.** | **.** | **.** | **.** | **.** | **.** | **.** | **.** | **.** |
| Hap_41 | T | | **.** | | **.** | **.** | **.** | **.** | **.** | **.** | **.** | **.** | **.** | **.** | **.** | **.** | **.** | **.** | **.** | **.** | **.** | **.** | **.** | **.** |
| Hap_42 | **.** | | **.** | | **.** | **.** | **.** | **.** | **.** | **.** | **.** | **.** | G | **.** | **.** | G | **.** | G | **.** | **.** | **.** | **.** | **.** | **.** |
| Hap_43 | **.** | | **.** | | **.** | **.** | **.** | **.** | **.** | **.** | **.** | **.** | **.** | **.** | T | **.** | **.** | G | **.** | **.** | **.** | **.** | **.** | **.** |
| Hap_44 | **.** | | **.** | | **.** | **.** | **.** | **.** | **.** | **.** | **.** | **.** | **.** | **.** | **.** | **.** | **.** | **.** | **.** | **.** | **.** | **.** | **.** | G |
| Hap_45 | **.** | | **.** | | **.** | **.** | **.** | **.** | **.** | A | **.** | **.** | **.** | **.** | T | G | **.** | **.** | **.** | **.** | A | **.** | **.** | **.** |
| Hap_46 | **.** | | **.** | | **.** | **.** | **.** | **.** | **.** | A | **.** | **.** | **.** | **.** | T | G | **.** | G | **.** | **.** | **.** | **.** | **.** | **.** |
| Hap_47 | **.** | | **.** | | **.** | **.** | **.** | **.** | **.** | A | **.** | **.** | **.** | **.** | T | G | **.** | G | **.** | **.** | A | **.** | **.** | **.** |
| Hap_48 | **.** | | **.** | | **.** | **.** | **.** | **.** | **.** | **.** | **.** | **.** | **.** | **.** | **.** | **.** | **.** | G | **.** | **.** | **.** | **.** | **.** | G |
| Hap_49 | **.** | | **.** | | **.** | **.** | **.** | **.** | **.** | **.** | **.** | **.** | **.** | **.** | **.** | G | **.** | **.** | **.** | **.** | **.** | **.** | **.** | G |
| Hap_50 | T | | **.** | | **.** | **.** | **.** | **.** | **.** | **.** | **.** | **.** | **.** | **.** | **.** | **.** | **.** | **.** | **.** | A | **.** | **.** | **.** | **.** |
| Hap_51 | **.** | | **.** | | **.** | **.** | **C** | **.** | **.** | A | **.** | **.** | G | **.** | **.** | **.** | **.** | **.** | **.** | **.** | **.** | **.** | **.** | G |
| Hap_52 | **.** | | **.** | | **.** | **.** | **C** | **.** | **.** | A | **.** | **.** | **.** | **.** | T | G | **.** | **.** | **.** | A | A | **.** | **.** | **.** |
| Hap_53 | **.** | | **.** | | **.** | **.** | **-** | **.** | **.** | **.** | **.** | **.** | G | **.** | **.** | G | **.** | **.** | **.** | **.** | **.** | **.** | **.** | G |
| Hap_54 | **.** | | **.** | | **.** | **.** | **.** | **.** | **.** | A | **.** | **.** | G | **.** | **.** | **.** | **.** | **.** | **.** | **.** | **.** | **.** | **.** | G |
