# Supplementary material for: Phylogeography and ecological niche modeling implicate multiple microrefugia of Swertia tetraptera during quaternary glaciations
Source: BMC Plant Biol. 2023 Sep 26;23:450. doi: 10.1186/s12870-023-04471-w (PMC10521563; doi:10.1186/s12870-023-04471-w)
Supplement: Supplementary file 5 — Supplementary Material 5 [file 12870_2023_4471_MOESM5_ESM.docx]

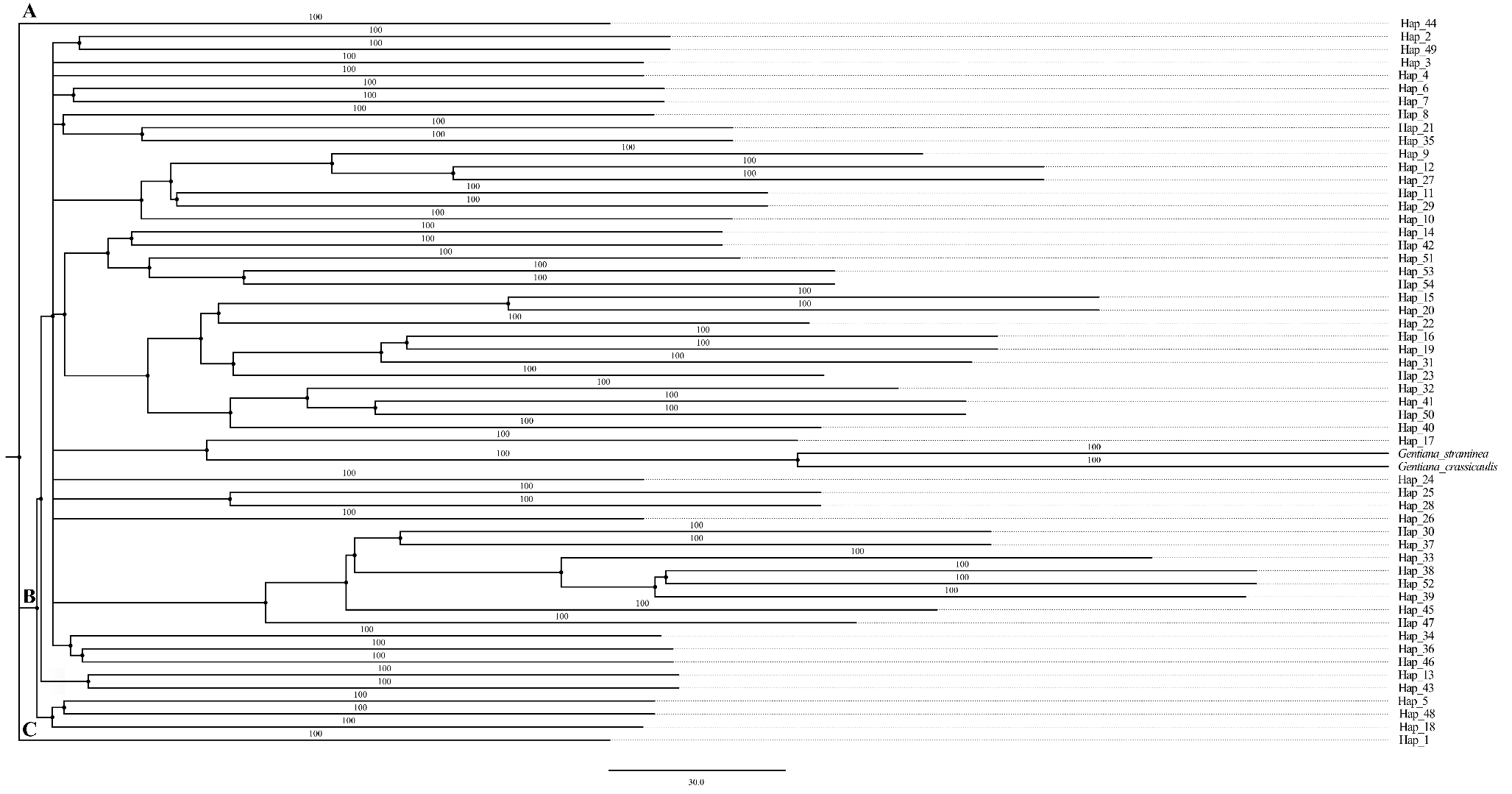


**Figure S1** Phylogenetic tree for 54 cpDNA haplotypes using maximum likelihood method. Bootstrap supports are shown above the branches, and only bootstrap supports >95 were listed.


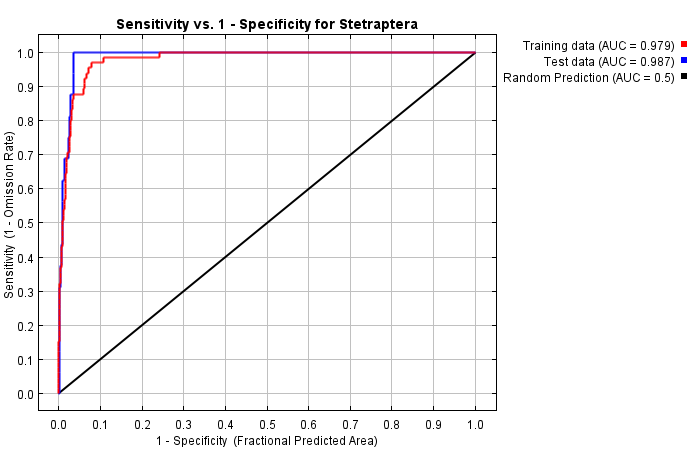


Figure S2 ROC curve of distribution of *S. tetraptera* predicted by MaxEnt model.





Figure S3 Divergence time of *Swertia* estimated using BEAST.
