# Supplementary material for: Phylogeography and ecological niche modeling implicate multiple microrefugia of Swertia tetraptera during quaternary glaciations
Source: BMC Plant Biol. 2023 Sep 26;23:450. doi: 10.1186/s12870-023-04471-w (PMC10521563; doi:10.1186/s12870-023-04471-w)
Supplement: Supplementary file 6 — Supplementary Material 6 [file 12870_2023_4471_MOESM6_ESM.pdf]

```
##############################################
# Model calibration using kuenm and Maxent #
##############################################

# Description
## The following script helps to perform the process of model calibration using
## Maxent through the R pacakge kuenm. Model calibration consists of a series of
## steps starting in the creation of several models and ending in the selection
## of parameter settings that produce the best models. To detect which ones are
## the models with best results a robust evaluation process must be performed. In
## the example, models are selected based on statistical significance, omission
## rates based on a predefined Error, and model complexity. The metrics used here,
## are partial ROC, omission rates, and AICc, in that order.

## The main processes are performed the package kuenm from GitHub. To install
## this package see instructions in https://github.com/marlonecobos/kuenm.


# loading needed package
# assuming that you installed kuenm, load it, if not installed see
# https://github.com/marlonecobos/kuenm for instructions
library(kuenm)

# set your working directory
setwd("C:/FC-RM")

# Candidate model creation
## check the functions help to understand arguments by typing '??kuenm', press enter and
check kuenm_cal documentation.

## preparing arguments (Change "YOUR/DIRECTORY" by your pertinent directory)
occ_joint <- "Mhenryi_joint.csv"
occ_tra <- "Mhenryi_train.csv"
M_var_dir <- "Environ_variables"
batch_cal <- "Candidate_models"
out_dir <- "Candidate_models"
reg_mult <- seq(0.1, 4, 0.1)
f_clas <- c("no.t")
args <- NULL # e.g., "maximumbackground=20000" for increasing the number of pixels in
the bacground or
            # note that some arguments are fixed in the function and should not be
changed
maxent_path <- "C:/FC-RM"      # where Maxent is
wait <- FALSE
```

```
run <- TRUE

## runing candidate models
kuenm_cal(occ.joint = occ_joint, occ.tra = occ_tra, M.var.dir = M_var_dir,
          batch = batch_cal, out.dir = out_dir, reg.mult = reg_mult, f.clas = f_clas,
          args = args, maxent.path = maxent_path, wait = wait, run = run)



# Candidate model evaluation and selection (YOU CAN DO THIS WHILE CANDIDATE MODELS ARE BEING CREATED)
## check the functions help to understand arguments

## preparing arguments (Change "YOUR/DIRECTORY" by your pertinent directory)
occ_test <- "Mhenryi_test.csv"
out_eval <- "Calibration_Results"
threshold <- 5
rand_percent <- 50
iterations <- 500
kept <- TRUE
selection <- "OR_AICc"
parallel_proc <- 10

## runing candidate models
cal_eval <- kuenm_ceval(path = out_dir, occ.joint = occ_joint, occ.tra = occ_tra,
                        occ.test = occ_test, batch = batch_cal, out.eval = out_eval,
                        threshold = threshold, rand.percent = rand_percent,
                        iterations = iterations, kept = kept, selection = selection,
parallel.proc = parallel_proc)

# CHECK RESULTS IN WORKING DIRECTORY AND IN THE OBJECT CREATED BEFORE.
```
